# *Eastern Equine Encephalomyelitis Virus* Infection in a Horse from California

**DOI:** 10.3201/eid0803.010199

**Published:** 2002-03

**Authors:** Robert P. Franklin, Hailu Kinde, Michele T. Jay, Laura D. Kramer, Emily-Gene N. Green, Robert E. Chiles, Eileen Ostlund, Stan Husted, Jonathan Smith, Michael D. Parker

**Affiliations:** * Humphrey, Giacopuzzi & Associates Equine Hospital, Somis, California, USA †University of California Davis, Davis, California, USA; ‡ California Department of Health Services, Sacramento, California, USA; § National Veterinary Service Laboratory, Ames, Iowa, USA; ¶ U.S. Army Medical Research Institute of Infectious Diseases, Fort Detrick, Maryland, USA

**Keywords:** Alphavirus, Encephalitis, Arbovirus, Togavirus, Reverse Transcription-Polymerase Chain Reaction, Vaccine

## Abstract

A yearling quarter horse, which was raised in southern California, received routine vaccinations for prevention of infection by *Eastern equine encephalomyelitis virus* (EEEV). One week later, severe neurologic signs developed, and the horse was humanely destroyed because vaccine-related encephalomyelitis was suspected. A final diagnosis of EEEV infection was established on the basis of acute onset of the neurologic signs, histopathologic and serologic testing, and isolation and molecular characterization of EEEV from brain tissue**.** The vaccine was extensively tested for viral inactivation. Nucleotide sequences from the vaccine and the virus isolated in the affected horse were also compared. In California, arboviral encephalomyelitides are rarely reported, and EEEV infection has not previously been documented. This report describes the occurrence of EEEV infection in the horse and the investigation to determine the source of infection, which was not definitively identified.

Eastern equine encephalomyelitis virus (EEEV) is a mosquito-borne virus in the family Togaviridae, genus Alphavirus. EEEV, Western equine encephalomyelitis virus (WEEV), and Venezuelan equine encephalomyelitis virus (VEEV) are related but genetically distinct alphaviruses. EEEV and VEEV are lethal in up to 90% of recognized equine cases, whereas WEEV is least virulent in horses, which have a mortality rate of approximately 40% (*1*). EEEV may also cause fatal encephalitis in humans (mortality rate 50%-75%) (*2*). In the United States, enzootic EEEV occurs mainly from New England to Florida and along the Gulf Coast, with rare reports of foci as far inland as Michigan and South Dakota (*3*). In North America, sylvatic populations and the mosquito Culiseta melanura maintain the virus in hardwood, salt-water swamp habitats. Large populations of this mosquito allow amplification of the virus by transmission among wild birds (*4*). In wild birds indigenous to North America, the infection is usually innocuous, whereas in pheasants, cranes, and emus, the disease is often lethal. C. melanura feeds almost exclusively on passerine birds; however, spillover of EEEV from the enzootic vector into several other mosquito species (e.g., Aedes spp.), which feed on tangential hosts such as humans and equines, may result in large epizootics with high mortality rates (*4*–*6*). Our paper describes a sporadic case of EEEV infection in a horse outside the known geographic range of this virus and the ensuing investigation to determine the source of exposure.

## Materials and Methods

### Case Report

In April 2000, a 14-month-old gelding quarter horse was seen at a veterinary referral hospital in southern California for sudden onset of quadraparesis and recumbency. The horse had no history of prior neurologic disease. He had been castrated approximately 90 days before the illness without complication. A multidose, multivalent vaccine containing formalin-inactivated EEEV and WEEV, influenza virus, and tetanus toxoid was administered to the affected horse and 27 stable mates 1 week before the onset of illness.

The horse appeared alert and healthy the night before onset of clinical signs. At 6:30 a.m. on April 21, he was found down in his stall and unresponsive to external stimuli. The referring veterinarian found a recumbent, comatose horse with spontaneous nystagmus and flailing, incoordinated movements. Initial therapy included intravenous corticosteroids, fluid therapy (including glucose to treat possible hyperkalemic periodic paralysis), and diazepam for intermittent seizures. The horse did not respond to therapy and was sent to the referral hospital.

On examination at the hospital, the horse was comatose with elevated heart and respiratory rates and a normal rectal temperature. A neurologic exam showed that pupillary light responses were absent bilaterally. Palpebral reflexes were present although weak. No organized motor movements occurred in response to stimuli. Initial emergency treatment consisted of intravenous fluids with dimethyl sulfoxide and flunixin meglumine. Cervical and skull radiographs were performed and were within normal limits. An atlanto-occipital cerebrospinal fluid tap was also performed, and no abnormalities were seen on gross observation. At this point, diffuse cortical disease was evident. Trauma appeared very unlikely, and an infectious process or toxicosis seemed more probable. Because of the grave prognosis, the owners elected to euthanize the horse. The carcass was sent to the Animal Health and Food Safety Laboratory System, San Bernardino Branch, School of Veterinary Medicine, University of California, Davis, for necropsy.

## Results

### Pathology

**Figure 1:**
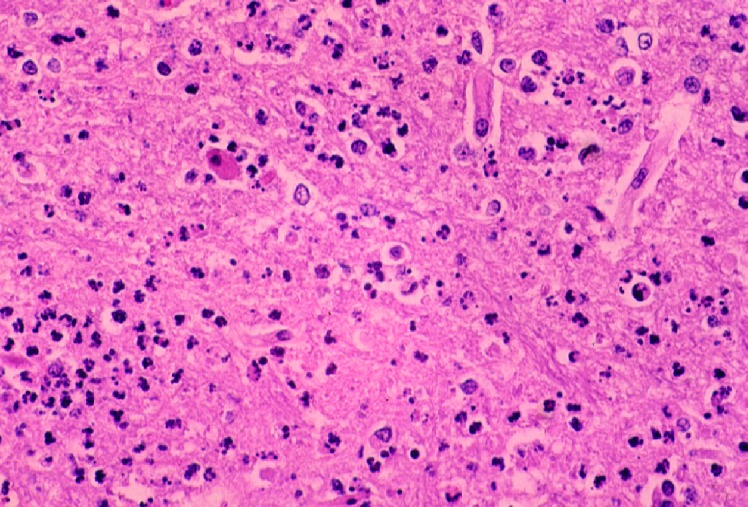
Photomicrograph of a section of the cerebral cortex from horse with *Eastern equine encephalomyelitis virus* infection. Note the dense neutrophilic response, vascular damage, and fibrin thrombi. Hematoxylin and eosine stain.

Results of gross necropsy examination were unremarkable except for markedly hemorrhagic bladder mucosa. Histologic examination revealed lesions mainly confined to the cerebral cortex, thalamus, hypothalamus, and anterior portion of the spinal cord (C_1_-C_4_). Lesions in the brain were characterized by a multifocal to diffuse neutrophilic response with gradual progression to mononuclear cell infiltrates in some areas. Vascular damage and fibrin thrombi were evident (Figure 1). Some blood vessels had swollen endothelium surrounded by a thick layer of mononuclear cells. A mild degree of meningitis was present, with pleocellular response containing mainly mononuclear and neutrophilic infiltrates. The neuropil showed fine vacuolation, indicating edema. Some axons were markedly shrunken. The remaining portion of the spinal cord was unremarkable. The urinary bladder had diffuse submucosal hemorrhages. The lung showed flooding of the alveoli with eosinophilic fluid. The remaining tissues were unremarkable.

### Virology

Portions of brain tissue were collected and sent for rabies testing at the local public health laboratory. Results were negative. Fresh, frozen brain tissues and serum were submitted to the National Veterinary Services Laboratories (NVSL). Tests for equine encephalomyelitides included virus isolation and serology for WEEV, EEEV, VEEV, *Equid herpesvirus 1* (EHV-1), and *West Nile virus* (WNV). For virus isolation, a 10% suspension of brain sample was prepared and injected into flasks of RK13, equine dermal, and Vero-MARU cells (Vero M). This cell line was obtained by NVSL at the 135th passage level from the Middle America Research Unit (MARU) as a multipurpose cell line for virus isolation in 1980 and has been maintained by the NVSL since that time. Additional brain suspension was injected intracerebrally into 16 suckling 4-day-old mice (from 2 litters). Cytopathic effects were observed in the RK13 and Vero M cells at 2 days after injection. Examination using electron microscopy of the RK13 cell culture fluids showed particles with morphologic features compatible with alphaviruses.

Virus preparations from both the cell culture supernatant and suckling mouse brains of mice that died were identified as EEEV by a complement fixation test with reference antisera. In that test, virus reacted strongly with EEEV antiserum and weakly or not at all with WEEV and VEEV antisera.

Serum collected from the yearling horse on April 21 before it died was tested for antibodies to EEEV, WEEV, and VEEV by hemagglutination inhibition (HI) and plaque reduction neutralization testing (PRNT). The serum had a HI titer of 20 against both EEEV and WEEV. HI antibodies to VEEV were not detected. In the PRNT, the serum neutralizing antibody titer versus EEEV was ≥100 but was undetectable against either WEEV or VEEV. The serum was also positive in an immunoglobulin (Ig) M-capture enzyme-linked immunosorbent assay for EEEV with a titer of ≥1000. Additional tests for antibodies to equine herpesvirus 1 and WNV were negative.

The Center for Vector-Borne Disease Research at the University of California, Davis (CVBDR), U.S. Army Medical Research Institute of Infectious Diseases (USAMRIID), and California Department of Health Services (CDHS) also received homogenized brain suspension material. Isolation of EEEV by mouse inoculation and cell culture supported NVSL’s findings. The isolate was further characterized by USAMRIID by reverse transcriptase-polymerase chain reaction (RT-PCR) testing and sequencing as described (*7*,*8*), but with primers listed in Table 1. A 1,165-nucleotide portion of the viral genome including parts or all of the E2, 6K, and E1 genes was determined. Sequencing of the isolate showed an 18-nucleotide difference (98.5% homology) from the reference PE6 EEEV strain. Comparison with sequences that have been submitted to GenBank indicated that the virus is a North American antigenic variety in subtype 1 of the taxonomic scheme recently proposed by Brault et al. (*9*).

**Table 1:** Reverse transcriptase-polymerase chain reaction (RT-PCR) primers used to sequence *Eastern equine encephalomyelitis virus* RNA

Title	Sense	Primer	Use	Region
9930	Forward	5´- GCGCTGCTTATTTTGCTGT -3	RT-PCR	E1
11582	Reverse	5´- ATTATGCGCTGCCTGTAGTGTTTA –3´	RT-PCR	E1
4043	Forward	5´- GTGCGCGGCGACATAACAAAGAG- 3´	RT-PCR	NSP3
4976	Reverse	5´- CCGGGGGTACAGTGCCAGAGAA –3´	RT-PCR	NSP3
10470	Forward	5´- ATGCCAAACTCATCATAGGTCCACT –3´	Sequencing	E1
10938	Forward	5´- GTATAGCCACCGTTGCCTACAAATC –3´	Sequencing	E1
11345	Forward	5´- CAGGCAGTGTATAAGGCTGTCTTAC –3´	Sequencing	E1
T3	Forward	5´- AATTAACCCTCACTAAAGGGA –3´	Sequencing	NSP3

### Field Investigation

Local, state, and federal agencies participated in a joint field and laboratory investigation to determine the source of infection. Four hypotheses were investigated to explain the occurrence of EEEV outside its usual range: 1) imported infection from an EEEV-endemic region, 2) autochthonous transmission by locally infected mosquitoes, 3) intentional inoculation of the horse with live EEEV by a person or by purposeful contamination of the vaccine and 4) incomplete inactivation of EEEV in a commercially inactivated viral vaccine.

#### Travel History

We found no evidence that this case was due to importation from an EEEV-endemic region. The horse was born in northern California and at 6 months of age was moved to southern California for training. The horse traveled as far east as Fort Worth, Texas, in July 1999 for showing purposes. The rest of the 1999 horse show season took place in southern California. The horse was last moved to a new stable (Farm A) in southern California during February 2000. He attended several shows in this area and as far east as Hurricane, Utah. A review of a list of participants at the horse shows recently attended showed no horses from EEEV-endemic areas and no reports of encephalomyelitis among other equine participants.

#### Surveillance for EEEV in Southern California

No evidence for autochthonous transmission of EEEV by local mosquito populations was found through surveillance in mosquitoes, sentinel chickens, wild birds, horses, and humans from the region. California has an extensive, long-established Arboviral Encephalitis Surveillance Program that is active from April through October each year (*10*,*11*). The program includes collection and testing of mosquito pools and sentinel chicken flocks for WEEV and *St. Louis encephalomyelitis virus* and surveillance for encephalomyelitis cases among equids, ratites (e.g., emus, ostrich)**,** and humans. Following recognition of the equine EEEV case, CDHS began including EEEV screening in its routine testing program.

Coincidentally, a sentinel chicken flock was located on Farm A. Sera submitted from this flock in April were retrospectively tested for EEEV antibody by indirect immunoassay and found to be negative. The flock remained seronegative for EEEV from May through October. In addition, mosquitoes were collected at Farm A and within a 5-mile radius with carbon dioxide traps. A total of 74 mosquitoes, including the *Culex* spp. *tarsalis*, *quinquefasciatus*, *erythrothorax*, and *stigmatosoma,* and *Culiseta particeps,* were collected in 23 trap nights during May. Only 8 of 74 mosquito species were *Culex tarsalis*, a known vector species of WEEV and a potential vector species of EEEV in California (*12*). Surveys for resting adult mosquitoes in barns and other buildings yielded no mosquitoes. All mosquito pools were tested and found negative by virus isolation in tissue culture. Routine biweekly testing of sentinel chicken flocks and mosquito pools throughout California until the end of October showed no further evidence of EEEV activity in the state.

Despite enhanced surveillance, additional cases of EEV infection in local animal and human populations were not identified. Surveillance for encephalitis cases in horses and humans was heightened in southern California after the equine case was recognized. Veterinarians were alerted statewide through a newsletter published by the California Department of Food and Agriculture, and the local health department issued a press release. Following the publicity, a veterinarian reported three horses with acute neurologic disease during mid-May at another ranch, Farm B, approximately 50 km from Farm A. Necropsy and serologic testing of these cases performed at the California Animal Health and Food Safety Laboratory System, San Bernardino Branch, showed EHV-1 as the likely cause of the outbreak at Farm B; no evidence of EEEV infection was found. In addition, a brown-headed cowbird (Molothrus ater*)* die-off at another horse ranch, Farm C, approximately 80 km from Farm A, was investigated. No laboratory evidence of EEEV infection was found in three dead cowbirds collected from Farm C, although the causes of their deaths were not determined.

No other horses at Farm A had encephalitis. To further assess potential equine exposures at Farm A, a serosurvey of 10 randomly chosen stable mates of the affected horse were tested for EEEV antibodies. The sample ranged in age from weanlings to elderly horses; each had been vaccinated with the multivalent vaccine against WEEV, EEEV, influenza viruses, and tetanus (Vaccine A) from the same lot on the same day as the case. These horses showed positive neutralizing antibody titers by PRNT ranging from <20 to 320; none had IgM antibodies to EEEV by an ELISA-capture test. Previous vaccination histories were not available for these horses or the case, but the findings in the stable mates were compatible with recent vaccination or the presence of maternal antibodies in younger horses without natural exposure.

#### Criminal Mischief

Although an intentional introduction of EEEV seemed highly unlikely, recent concerns about bioterrorism made this an important possibility to consider. We found no evidence of purposeful contamination of the vaccine or intentional inoculation of the horse. EEEV is not readily obtainable. Furthermore, no motive for such an act was found.

#### Vaccine Studies

An extensive evaluation of the final hypothesis, residual live EEEV in the vaccine, could not eliminate Vaccine A as the source of infection. The farm manager ordered the multivalent EEEV, WEEV, influenza viruses, and tetanus toxoid vaccine by mail from an out-of-state vendor and stored the vials at 6° C in a refrigerator at Farm A. The vaccine was a commercial, four-way, multidose product that was administered intramuscularly by farm personnel. The viruses in the vaccine were formalin-inactivated, adjuvant-type, and of tissue-culture origin.

#### Virus Isolation

Three unused vials and one partially used vial of Vaccine A were found in the refrigerator at Farm A. CDHS and CVBDR attempted virus isolation by mouse inoculation and cell culture by using the residual vaccine from Farm A. One-day-old mice were inoculated by either intraperitoneal or intracranial injections of the vaccine and were monitored for 18 days. Live EEEV was not isolated from any of the vials. Additionally, the Center for Veterinary Biologics Laboratory conducted safety tests on stored vaccine from the same lot as Vaccine A, which was available because of licensing procedures that require samples from each lot to be retained. Virus isolation attempts on these samples were also negative by cell culture and wet chick inoculations.

#### Molecular Comparison of Horse and Vaccine Strains

**Figure 2:**
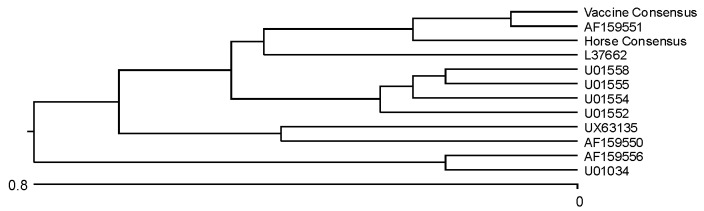
Phylogram based on nucleotide comparison from the E1 region of a horse infected with *Eastern equine encephalomyelitis virus*.

EEE viral RNA was extracted from the horse isolate and passaged once via BHK (baby hamster kidney) cell culture at CVBDR and directly from the residual vaccine and amplified and sequenced in two separate regions of the genome according to previously published protocols (*7*,*8*). The structural E1 region was amplified and compared with several other published EEEV E1 sequences in GenBank; 1,100-nucleotide sequences of the horse isolate and Vaccine A strain were compared with each other as well as with the 10 most closely matched published sequences in GenBank. The conclusions from this laboratory’s study were very similar to the initial gene sequencing of the horse isolate by USAMRIID. Table 2 illustrates a comparison of each GenBank sequence to the sequence of the horse isolate. The phylogram is depicted in Figure 2.

**Table 2:** Comparisons of 1,100 nucleotide sequences of the horse virus isolate, Vaccine A virus strain, and selected GenBank isolates of the structural unit E1

GenBank accession no.	Strain/isolate	Mutations	% Match
Horse		0	100
Vaccine		5	99.5
AF159551	LA50	7	99.4
L37662	PE6 vaccine	11	98.9
U01552	Decuir	13	98.5
U01558	Tenbroeck	15	98.6
U01554	NJ/60	15	98.6
AF159556	FL96-14834	17	98.5
X63135	ssp. N. Am. Variant	18	98.4
AF159550	MA38-Mass	19	98.3
U01555	ME771332	19	98.2
U01034	82V2137	19	98.3

**Figure 3:**
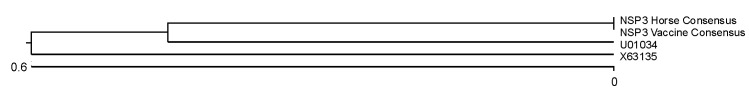
Phlyogram based on nucleotide comparison from the NPS3 (nonstructural) region of a horse infected with *Eastern equine encephalomyelitis virus*.

The NSP3 (nonstructural) region was also amplified and cloned to check for variability within Vaccine A and in the horse viral isolate, as well as to compare with published EEEV NSP3 sequences in GenBank. Four horse and five vaccine viral RNA clones were sequenced and analyzed. Of the 508 nucleotides in each fragment, only one nucleotide difference was evident among the cloned vaccine sequences, and only one was found among the four sequences from horse isolates. These differences could be a result of taq polymerase errors. The consensus sequences for both the vaccine and the horse EEEVl RNAs were compared with each other and with the only two EEEV GenBank sequences that came up in a BLAST search (Table 3, Figure 3).

**Table 3:** Comparisons of 508 nucleotide sequences of the horse virus isolate, Vaccine A virus strain, and selected GenBank isolates of the nonstructural unit NSP3

GenBank accession no.	Strain/isolate	Mutations	% Match
Horse		0	100
Vaccine		0	100
X63135	ssp. N. Am. Variant	5	99.0
U01034	82V2137	5	99.0

## Discussion

The identification of EEEV in a horse in California was unprecedented and clearly represented a potential human and animal health threat. In other areas of the country, equine epizootics have been recognized as precursors to human disease (*13*,*14*). The rapid recognition and reporting of the case permitted an extensive investigation into the source of exposure.

 Several factors must be met to sustain epidemics, including virulent viruses, adequate vectors, neighboring intermediate hosts, and populations of susceptible horses and people (*5*,*15*). Such isolated cases as the one mentioned are sure to increase veterinary attention to the possibility of neurologic patients having EEEV infections, as well as elevating public awareness of the disease and methods of prophylaxis.

A diagnosis of EEEV infection was made on the basis of the rapid clinical onset of neurologic signs, compatible histopathologic and serologic findings, and isolation and molecular characterization of EEEV from brain tissue. Several neurologic conditions were considered in the differential diagnosis, including other viral encephalomyelitides (rabies, Aujesky disease, Borna disease, EHV-1 myeloencephalopathy, WEEV, and WNV encephalomyelitis), bacterial meningitis, listeriosis, leukoencephalomalacia, lead poisoning, equine protozoal myeloencephalitis, nigropallidal encephalomalacia, botulism, and verminous encephalitis.

California’s Arboviral Encephalitis Surveillance Program is among the most comprehensive in the United States. The jurisdiction where the horse was stabled participated in the program, and a sentinel chicken flock was located adjacent to the farm. In this case, locally infected mosquitoes were apparently not the source of exposure. Furthermore, there was no evidence of spread from the infected horse to the local mosquito populations based on mosquito pool and sentinel chicken flock testing throughout the year. The likelihood of EEEV’s having become established in California following this isolated equine case is remote but still important to monitor because of the public health implications. First, the primary vector of EEEV in North America, *C. melanura*, is not known to occur in California (*16*). In addition, our equine case was diagnosed in April, when mosquito populations are low in southern California; particularly the vector species known to feed on both birds and horses. Second, this case had a rapid clinical course, with euthanasia in <24 hours after onset of clinical signs. Since horses are known to have a short viremia (1 to 3 days’ duration) it is unlikely that any mosquitoes acquired the infection from the horse during this short time period. However, if vector abundance were increased, this horse would have had the potential to amplify the virus (*5*). Incidental infections could have occurred among barn personnel and susceptible horses at Farm A and nearby locations by transmission from mosquitoes that acquired the infection from the case. Of even greater concern, competent vectors could then spread the disease further by feeding on susceptible wild bird populations, potentially establishing an enzootic cycle in southern California.

After we excluded disease by natural infection, bioterrorism, and importation, incomplete formalin inactivation of the EEEV in the vaccine had to be considered a likely possibility. Previous reports of residual virus in formalin-inactivated vaccines exist. Documented outbreaks due to *Poliovirus* (PV*),* *Foot-and-mouth disease virus,* and VEEV have been directly related to the use of formalin-inactivated vaccines (*17*–*19*). Attempts to isolate live EEEV from residual and stored vaccine were unsuccessful. However, this does not eliminate the possibility that the horse received live virus with its immunization. If inactivated viruses existed in the vaccine, they were likely present in undetectable levels during vaccine development and testing. Additionally, the live viruses were probably distributed sporadically throughout the vaccine lot, allowing for only an isolated recognized case. The situation could also be analogous to the 1955 “Cutter inactivated poliovirus incident,” when children became infected with PV after vaccination and follow-up investigation disclosed that several lots of Salk PV vaccine contained live PV, despite being produced with formalin inactivation in full compliance with federal regulations (*19*). In the PV vaccine example, live virus was not uniformly distributed in that vaccine lot (*20*).

We further explored the hypothesis of residual live virus in the vaccine through molecular epidemiologic studies. Similar studies were used to examine the role of the VEEV vaccine in the 1967-1972 VEEV pandemic in Central America (*21*). Unfortunately, the North American variety of EEEV is the most genetically homologous of the alphaviruses and therefore the least conducive to molecular comparison of strains (*9*,*22*). In our study, the greatest nucleotide homology in the E1 region was among the horse virus isolate, Vaccine A virus, and the LA50 virus strain (Figure 2). Differences among sequences from Vaccine A EEEV and the horse viral isolate in the E1 region might represent mutations that occurred when virus passed through various hosts (horse brain/BHK cell culture/1) or genetic variants within the vaccine strain. However, we concluded on the basis of the limited number of clones analyzed that there were few to no other EEEV subclones in the horse viral isolate or vaccine virus. The NSP3 region proved to be more highly conserved and therefore less conclusive. Also, very few EEEV sequences that included the nonstructural regions have been published in GenBank, so comparison was limited. Regardless, the Vaccine A EEEV appears to be closely related to the horse viral isolate; thus, the possibility of live virus in the formalin-treated vaccine infecting the horse remains.

We are unaware of any reports of problems with this vaccine lot, despite notification of the manufacturer and other state veterinarians. If Vaccine A or portions of the lot contained live virus, many exposed horses may not have been susceptible because of previous immunization or presence of maternal antibodies. In addition, cases may have been unrecognized or unreported. If Vaccine A was the source of infection for this case or other cases, it was probably a rare event.

 A definitive source of infection may never be revealed in this case. However, the case illustrates the need to maintain awareness that EEEV can occur outside its normal geographic boundaries; it also underscores the importance of prompt diagnosis, reporting, and surveillance for arboviral encephalomyelitides.
